# Mast Cells Modulate Antigen-Specific CD8^+^ T Cell Activation During LCMV Infection

**DOI:** 10.3389/fimmu.2021.688347

**Published:** 2021-06-14

**Authors:** Yana Hackler, Frank Siebenhaar, Max Löhning, Marcus Maurer, Melba Muñoz

**Affiliations:** ^1^ Dermatological Allergology, Allergie-Centrum-Charité, Department of Dermatology, Venereology and Allergology, Charité - Universitätsmedizin Berlin, Corporate Member of Freie Universität Berlin and Humboldt-Universität zu Berlin, Berlin, Germany; ^2^ Experimental Immunology and Osteoarthritis Research, Department of Rheumatology and Clinical Immunology, Charité - Universitätsmedizin Berlin, Corporate Member of Freie Universität Berlin and Humboldt-Universität zu Berlin, Berlin, Germany; ^3^ Pitzer Laboratory of Osteoarthritis Research, German Rheumatism Research Center (DRFZ), A Leibniz Institute, Berlin, Germany; ^4^ Clinician Scientist Program, Berlin Institute of Health at Charité - Universitätsmedizin Berlin, Berlin, Germany

**Keywords:** mast cells, CD8^+^ T cells, viral infection, immunosuppression, dendritic cells

## Abstract

Mast cells (MCs), strategically localized at mucosal surfaces, provide first-line defense against pathogens and shape innate and adaptive immune responses. Recent studies have shown that MCs are involved in pathogenic responses to several viruses including herpes simplex viruses, dengue virus, vaccinia virus and influenza virus. However, the underlying mechanisms of MCs in the activation of CD8^+^ T cells during viral infections are not fully understood. Therefore, we investigate the role of MCs in the development of virus-specific CD8^+^ T cell responses using the well-characterized murine lymphocytic choriomeningitis virus (LCMV) model and the transgenic MasTRECK mice that contain the human diphtheria toxin receptor as an inducible MC-deficient model. Here, we report that MCs are essential for the activation and expansion of virus-specific CD8^+^ T cells. After MC depletion and subsequent intradermal LCMV infection, the CD8*^+^* T cell effector phenotype and antiviral cytokine production were impaired at the peak of infection (day 8 p.i.). Importantly, MC-deficient mice were unable to control the infection and exhibited significantly higher viral loads in the spleen and in the ear draining lymph nodes compared to that of wild type control mice. In the absence of MCs, dendritic cell (DC) activation was impaired upon LCMV infection. In addition, type-I interferon (IFN) levels in the serum and in the spleen of MC-deficient mice were reduced during the first days of infection. Interestingly, depletion of MCs after intradermal LCMV infection did not impair virus-specific CD8^+^ T cell expansion, activation or antiviral cytokine production. In summary, our results indicate that MCs play a pivotal role in the activation and antiviral functions of CD8^+^ T cells through proper DC activation. A better understanding of the impact of MCs on CD8^+^ T cell responses is mandatory to improve antiviral immune responses.

## Introduction

Mast cells (MCs) are long-lived immune cells distributed throughout nearly all tissues and particularly close to the skin and mucosa (1). MCs can quickly respond to invading pathogens and initiate immune responses due to their location and the expression of a wide spectrum of pattern recognition receptors (2–4). In addition, MCs sense stress and tissue damage *via* receptors of danger-associated molecular patterns (5–7). Furthermore, MCs can release a plethora of immune mediators including cytokines, chemokines, proteases, and antimicrobial peptides, which allow them to activate both immune and non-immune cells (8, 9). Thus, MCs can be considered a bridge between innate and adaptive immune responses (10). Several studies have shown that MCs play a protective role during bacterial, fungal and parasitic infections (11–16). In addition, increasing evidence using experimental infection models in mouse and human cell lines have revealed novel insights into the role of MCs in viral infections.

MCs can directly sense viruses (2) and can also be activated by inflammatory mediators produced during viral infections (17). Depending on the mechanism of viral recognition, MCs release immune mediators through degranulation or *de novo* cytokine and chemokine production (18). MCs have shown to modulate the course of cytomegalovirus, vaccinia virus, influenza virus, epstein barr virus and dengue virus infections (19–22). Moreover, MCs mediate the recruitment of short-lived effector CD8^+^ T cells into the lung in a CCL-5 dependent manner after cytomegalovirus infection (19). Antimicrobial peptides produced by MCs such as cathelicidin exert antiviral properties against vaccinia virus infection as shown by increased viral loads in infected MC-deficient mice compared to infected wild type animals (23). MCs not only mediate recruitment of cytotoxic cells such as NK cells, NKT cells, CD8^+^ T and γδ T cells (24, 25) but also contribute to viral clearance after dengue virus infection (26). Similarly, MC-deficient mice exhibited increased clinical severity and mortality with elevated virus titers compared to wild type mice after a HSV-2 infection (27).

Recent studies show that DC-MC interactions have a strong impact in the modulation of DC migration, activation and function (28–30). In addition, molecular transfers of major histocompatibility complex class II (MHCII) proteins between MCs and DCs enhanced T cell priming efficiency (31). MCs not only induce DC migration but also enhance DC maturation *in vitro*, antigen uptake, and cross-presentation (28, 32). In addition to direct MC-DC communication, a recent study show that MC granules and exosomes are able to promote DC maturation (33). MCs have been shown to induce the activation and migration of antigen-presenting cells from the skin. MC-deficient Kit^W-sh/W-sh^ or TNF(-/-) mice showed significantly reduced migration of airway DCs to local LNs 24 h after intranasal challenge with FITC-OVA in a model of contact hypersensitivity to FITC (34). In addition, activated mast cells were shown to alter the pulmonary micromilieu and induce antigen uptake, activation and migration of DCs (35).

Despite the increasing evidence for the critical role of MCs in immune responses and their protective role in viral infections, the underlying mechanisms are still not completely understood. Here, we report that MCs are crucial for the activation, expansion and function of virus-specific CD8^+^ T cells. Accordingly, MC-deficient mice were not able to control the infection and exhibited high viral loads in the spleen and in the ear draining lymph nodes (ear-dLNs). In the absence of MCs, DC activation was impaired and type-I IFN levels were reduced. Furthermore, MC-deficient mice exhibited diminished chemokine concentrations that led to decreased recruitment of DCs to secondary lymphoid organs. Thus, our findings indicate that MCs are essential for the development of antigen-specific CD8^+^ T cells responses during viral infections.

## Materials and Methods

### Mice

C57BL/6 (WT) and MasTRECK mice on C57BL/6 background (36) were bred in the animal facility at Charité, Berlin under specific pathogen-free conditions. C57BL/6 (WT) were used for peritoneal mast cell and DC isolation. For mast cell depletion, C57BL/6 (WT) and MasTRECK mice received 250 ng of diphtheria toxin intraperitoneally during five consecutive days. All animal experiments were performed at the Charité, Berlin in accordance with the German law for animal protection and approved by the Landesamt für Gesundheit und Soziales of Berlin (LaGeSO approval number G0078/17).

### Virus, Measurement of Viral Titers and Inoculation of Mice

LCMV-WE strain was propagated on L-929 cells. LCMV stocks and viral titers in the spleen and the ear-dLNs were titrated by standard immunofocus assays on MC57G cells as described previously (37). In brief, MC57G cells were plated with organ homogenates or virus stock dilutions and subsequently overlaid with 2% methylcellulose. After 48 h of incubation at 37°C, the confluent monolayer of cells was fixed with 4 % formaldehyde, permeabilized with 1 % Triton X-100 (v/v) and stained with antibodies against LCMV nucleoprotein (VL-4). After a secondary staining step with peroxidase-conjugated anti-rat IgG antibody (Jackson), foci were developed by 20 min incubation with OPD substrate (0.1 mol/L Na_2_HPO_4_, 0.5 mol/L citric acid, 0.03 % H_2_O_2_, and 20 mg o-phenylenediamine dihydrochloride). Mice were intradermally infected on the ventral side of the ear pinna with 9*10^4^ PFU of LCMV-WE in 10 µL PBS.

### Preparation of Ear Cell Suspensions

Ears were splitted in two layers, cut into small pieces and subsequently placed into fleshly prepared digestion medium (915 µL RPMI, 40 µL FCS (4 %), 0.5 µL DNase (40 µg/mL) (Roche), 35 µL Liberase™ (10 mg/mL) (Roche) and 10 µL hyaluronidase (50 mg/mL). After incubation (60 min, 37°C, 1400 rpm) undigested tissue was removed using a 30 µm cell strainer and the cells were washed (10 min, 4°C, 300 g) in PBS/BSA (0.5 % w/v). Afterwards the single cell suspension was used for flow cytometry staining.

### Flow Cytometry Analysis

Surface receptor staining and intracellular cytokine staining procedures have been described previously (38). The LCMV-specific CD8+ T cell response to the dominant glycoprotein-derived epitope GP33 and the nucleoprotein-derived epitope NP396 were assessed by MHC class I tetramer staining as described previously (39).

Surface receptor staining was performed in single cell suspensions of ears, ear-dLNs and the spleen after homogenization using mechanical disruption through a 70 μm cell strainer. FACS analysis included surface marker stainings for anti-CD4 (GK1.5), anti-CD45 (30-F11), anti-KLRG1, anti-CD127 (IL-7R), anti-CD62L, anti-CXCR3 (CXCR3-173), anti-CCR2 (SA203G11), anti-MHCII (M5/114.15.2), anti-CD11c (N418), anti-CD11b (M1/70), anti-CD8 (53-6.7), anti-CD80 (16-10A1), anti-CD86 (GL1), anti-Ly6C (HK1.4), anti-Ly6G (1A8) anti-B220 (RA3-6B2), anti-F4/80 (BM8) and anti-Siglec H (551) (BioLegend). Zombie-Aqua (BioLegend) was used as a live/dead discrimination marker. Rat IgG1 (R3-34) and rat IgG2a (R35-95) isotype control antibodies (BD Biosciences) were used at the same concentrations as the respective cytokine antibodies.

For the analysis of intracellular cytokines, cells were restimulated with GP33 (10^-6^ mol/L) and NP396 (10^-6^ mol/L) (Neosystem) for infected animals or with PMA (5 ng/ml) and ionomycin (500 ng/ml; Sigma) for uninfected mice. 5 μg/mL brefeldin A (Sigma-Aldrich) was added after 30 minutes. After 3 h, surface marker stainings were performed and cells were subsequently fixed with 2 % formaldehyde (Merck). Later on, cells were stained with the following rat anti-mouse cytokine antibodies or isotype control antibodies in permeabilization buffer containing 0.05% saponin (Sigma-Aldrich): anti-IFN-γ (XMG1.2), anti-TNF-α (MP6-XT22) and anti-IL-10 (JES5-16E3) in permeabilization buffer containing 0.05 % saponin (Sigma-Aldrich). Rat IgG1 (R3-34) and rat IgG2a (R35-95) isotype control antibodies (BD Biosciences) were used at the same concentrations as the respective cytokine antibodies. Flow cytometry analysis was performed in BD FACS Canto II. The gating strategy for flow cytometry analysis of immune subsets in the spleen, ear-dLNs and ears is shown in **Figure S1**. After gating on live cells and subsequently on CD45^+^ cell, neutrophils (Ly6G^+^ CD11b^+^), macrophages (F4/80^+^ CD11b^+^) DC, (MCHII^+^ CD11c^+^) and inflammatory monocyte (LyC6^+^ CD11b^+^) were gated. For CD8^+^ T cells subsets, cells were gated on CD8^+^ T cells and subsequently, on KLRG-1^+^ IL-7R^-^ or CD44^+^ CD62L^-^ or CXCL3^+^ for activated CD8^+^ T cells, GP33-Tetramer^+^ and NP396-Tetramer^+^ for LCMV-specific CD8^+^ T cells and IFN-γ^+^ and IL-10^+^ for cytokine production (**Figure S2**). For lymphocytic DCs, analysis gates were set on CD8^+^CD11b^-^ cells (**Figure S3A**) and for pDC, after excluding CD3^+^ CD19^+^ and CD11b^+^ cells were gated on B220^+^ and lastly on Siglec-H^+^ (**Figure S3B**).

### LCMV Infection Model In Vitro

For peritoneal MC isolation, the abdominal skin of mice was washed with 70 % ethanol. The peritoneum was exposed by a 1‐cm midline abdominal incision, and 4.0 mL of sterile, pyrogen‐free, 0.9 % NaCl and 4.0 mL of air were injected into the peritoneal cavity via a 22‐gauge needle. The abdomen was massaged gently for ∼3 min and the peritoneal fluid was recovered via a 22‐gauge needle. Peritoneal MCs show a purity ≥ 95% (depicted by FcϵRI+ c-Kit+) after isolation in the flow cytometry analysis (**Figure S4A**). Subsequently, peritoneal MCs were then wash with PBS, infected with LCMV-WE at MOI 5 and cultured in RPMI 1640 plus GlutaMax-I supplemented with 10 % (vol/vol) FCS (Gibco; Life Technologies), penicillin (100 U/mL; Gibco; Life Technologies), streptomycin (100 μg/mL; Gibco; Life Technologies), and β-mercaptoethanol (50 ng/mL; Sigma-Aldrich) for 24h. Cells were harvested and stained for flow cytometry analysis. Supernatants were collected for cytokine and chemokine detection.

### Cytokine and Chemokine Analysis in Supernatants

The concentrations of cytokines and chemokines were determined in supernatants of 2x10e5 splenocytes and cells from ear draining lymph nodes cultured in a 96 U-bottom well plate in 200 µL RPMI complete medium for 24 h and using a magnetic bead based multiplex ELISA (LEGENDplex™-BioLegend) and the chemokine 26-Plex Mouse ProcartaPlex™ Panel 1 (Invitrogen) according manufacturer’s instructions.

### Immunohistochemistry

Paraffin sections (5 µm) were deparaffinized as follows: 2 x 10 min in xylol, 2 x 3 min in absolute ethanol, 2 x 3 min in 96 % ethanol, 1 x 3 min in 70 % ethanol, 3 x 5 min in deionized water and 3 x 5 s in TBS buffer (Tris-Base (7,4 mmol/L), Tris-HCl (43,5 mmol/L), NaCl (150 mmol/L), pH = 7.5). For MC staining, the sections were incubated with Avidin-FITC (BioLegend) for 15 min in the dark. After washing (3 x 3 min with TBS) the sections were embedded with Fluoromount-G™ containing DAPI (Thermo Fisher Scientific) and dried for 24 h.

### Statistical Analysis

GraphPad Prism (v8.0) software was used for data analysis. Statistical significance was determined by Student’s t-test (unpaired two-tailed) for all figures when not indicated different. More than two groups were compared via one-way ANOVA with Bonferroni’s post test for multiple comparisons. P = 0.01 to 0.05 was considered statistically significant (*), p = 0.001 to 0.01 as very significant (**), and p < 0.001 (***) as extremely significant, ns, not significant.

## Results

### Mast Cells Are Crucial for Immune Cell Recruitment to the Site of LCMV Infection

**Figure 1:**
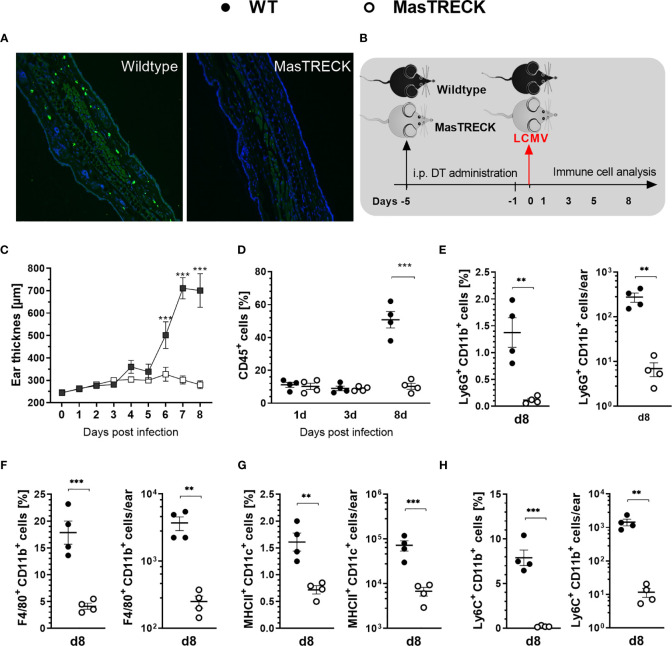
Mast cells are crucial for immune cell recruitment to the site of LCMV infection. **(A)** Immunohistochemistry analysis of paraffin ear sections using Avidin-FITC and DAPI to assess MCs and cell nuclei, respectively after DT treatment. **(B)** Schematic experimental layout to analyze immune responses in intraperitoneally treated WT and MasTRECK mice for 5 consecutive days with 250 ng DT followed one day after by an intradermal LCMV infection into the ventral side of the ear pinna. **(C)** Ear thickness daily measured using a caliper during 8 days after LCMV infection. **(D)** Frequencies of CD45^+^ cells in the ear of WT and MasTRECK mice on day 1, 3 and 8 post LCMV infection analyzed by flow cytometry. Frequencies and absolute numbers of **(E)** neutrophils (Ly6G^+^CD11b^+^), **(F)** macrophages (F4/80^+^CD11b^+^), **(G)** DCs (MHCII^+^CD11c^+^) and **(H)** inflammatory monocytes (Ly6C^+^CD11b^+^) in the ear of WT and MasTRECK mice assessed by flow cytometry on day 8 post LCMV. All experiments were performed at least twice, and each experimental group included n ≥ 4. Data are representative and expressed as mean ± SEM. Statistically significant differences are analyzed by *t-* test and indicated as follows: **p < 0.01, ***p < 0.001.

In order to investigate the role of MCs in the development of virus-specific CD8+ T cell responses, we used transgenic MasTRECK mice that contain the human diphtheria toxin (DT) receptor under the control of an intronic enhancer that is essential for *Il4* gene transcription in MCs but not in other cell types (36). After five days of DT i.p. application into both wildtype (WT) and MasTRECK mice, MCs were depleted in the skin of MasTRECK mice but not in that of WT mice (**Figure 1A**). The frequencies of different immune cell subsets in the spleen, ear draining lymph nodes (ear-dLNs) and ears were comparable between uninfected WT and MasTRECK mice after MC depletion (**Figures S1** and **S5A–E**). Basophils are concomitantly depleted with MC after DT administration and are therefore absent in MasTRECK mice during the course of LCMV infection including the last time point of analysis (day 8 p.i.) (36). One day after the last DT treatment, WT and MasTRECK mice were intradermally infected with LCMV on the ventral side of the ear pinna (**Figure 1B**). WT mice displayed a significant increase in ear thickness from day 6 to day 8 post infection compared to infected MasTRECK mice (**Figure 1C**). Furthermore, frequencies of haematopoietic CD45^+^ cells in the ear of WT mice were markedly increased compared to that of infected MasTRECK mice at the peak of infection (day 8 p.i.) (**Figure 1D**). In addition, infected MasTRECK mice displayed significantly reduced frequencies and absolute numbers of neutrophils, macrophages, DCs, and inflammatory monocytes compared to that of infected WT mice, suggesting that MCs are crucial for the recruitment of immune cells to the site of LCMV infection (**Figures 1E–H**).

### Mast Cells Are Key Players in the Expansion and Recruitment of Virus-Specific CD8*^+^* T Cells

**Figure 2:**
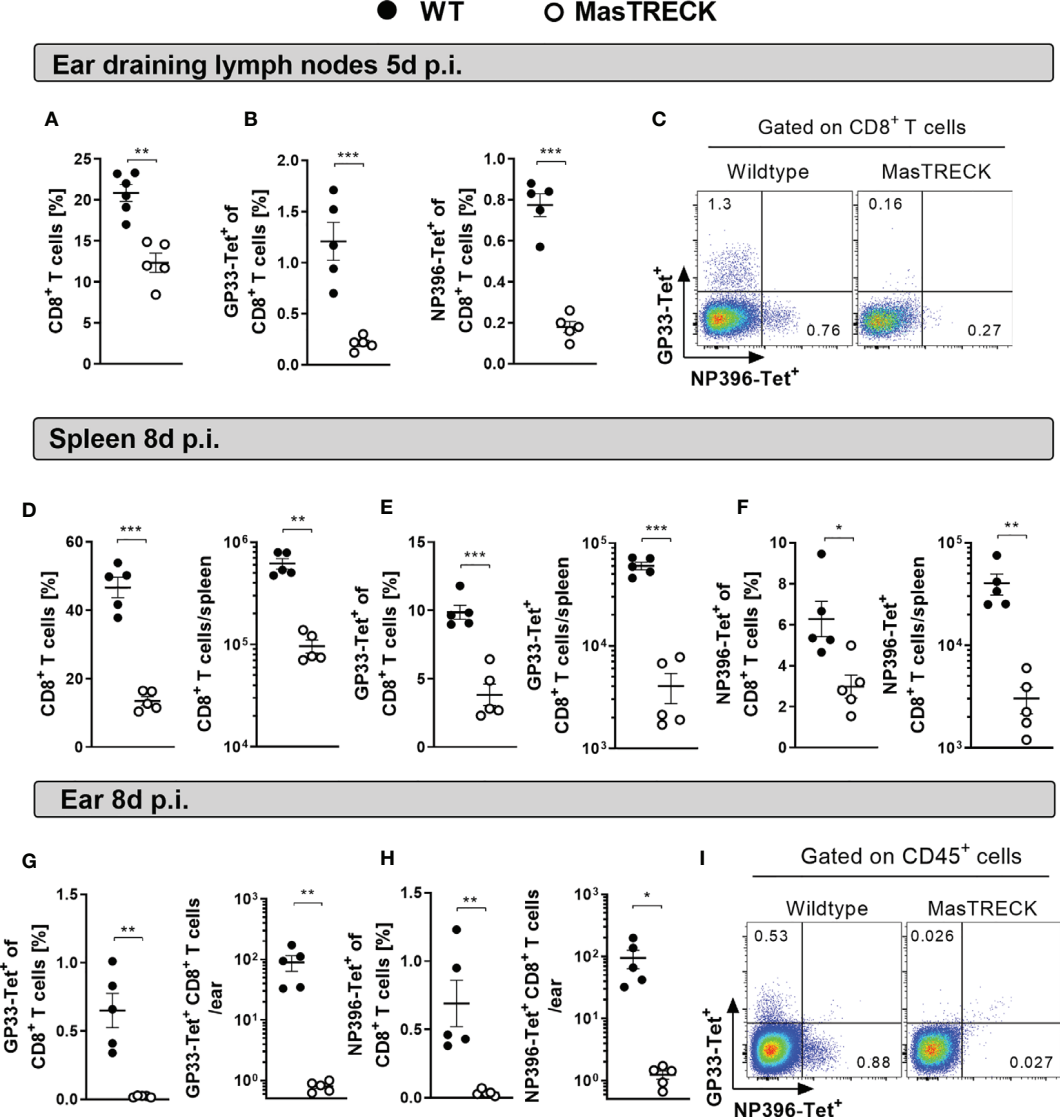
Mast cells are key players in the expansion and recruitment of virus-specific CD8^+^ T cells. **(A)** Frequencies of CD8^+^ T cells, **(B)** GP33-Tetramer^+^ and NP396-Tetramer^+^ CD8^+^ T cells in the ear-dLN as well as **(C)** representative FACS plots from WT and MasTRECK mice at day 5 post intradermal LCMV infection. Frequencies and absolute numbers of **(D)** CD8^+^ T cells, **(E)** GP33-Tet^+^ and **(F)** NP396-Tet^+^ CD8^+^ T cells in the spleen of WT and MasTRECK mice at day 8 post LCMV infection. Frequencies and absolute numbers of **(G)** GP33-Tet^+^ and **(H)** NP396-Tet^+^ cells in the ear of WT and MasTRECK mice as well as **(I)** representative FACS plots at day 8 post LCMV infection analyzed by flow cytometry. All experiments were performed at least twice, and each experimental group included n ≥ 4. Data are representative and expressed as mean ± SEM. Statistically significant differences are analyzed by *t-* test and indicated as follows: *p < 0.05, **p < 0.01, ***p < 0.001.

We then examined LCMV-specific CD8^+^ T cell responses in WT and MasTRECK mice treated with DT for 5 days followed by intradermal infection with LCMV on the ventral side of the ear pinna (**Figure 1B**). Frequencies of total CD8*^+^* T cells (**Figure 2A**) and CD8^+^ T cells specific for the dominant LCMV glycoprotein epitope GP33 and nucleoprotein epitope NP396 were markedly reduced in the ear-dLNs of MasTRECK mice compared to that of WT animals at day 5 post infection (**Figures 2B, C**). At this early time point, virtually no virus-specific CD8*^+^* T cells were observed in the spleen of both groups of infected animals (**Figure S6**). At the peak of infection, on day 8, frequencies and absolute cell numbers of CD8*^+^* T cells (**Figure 2D**) as well as of GP33- and NP396-tetramer^+^ CD8^+^ T cells were markedly reduced in the spleen of MasTRECK mice compared to that of WT animals (**Figures 2E, F**). Accordingly, GP33- and NP396-tetramer^+^ CD8*^+^* T cell frequencies and absolute numbers were also diminished in the infected ear of MasTRECK mice compared to that of WT animals at day 8 post infection (**Figures 2G–I**). Taken together, these data indicate that the expansion of virus-specific CD8^+^ T cells in the secondary lymph organs and their recruitment to the site of the infection are impaired in the absence of MCs.

### Mast Cell Deficient Mice Show Impaired CD8*^+^* T Cell Effector Phenotype and Antiviral Cytokine Production After Infection

**Figure 3:**
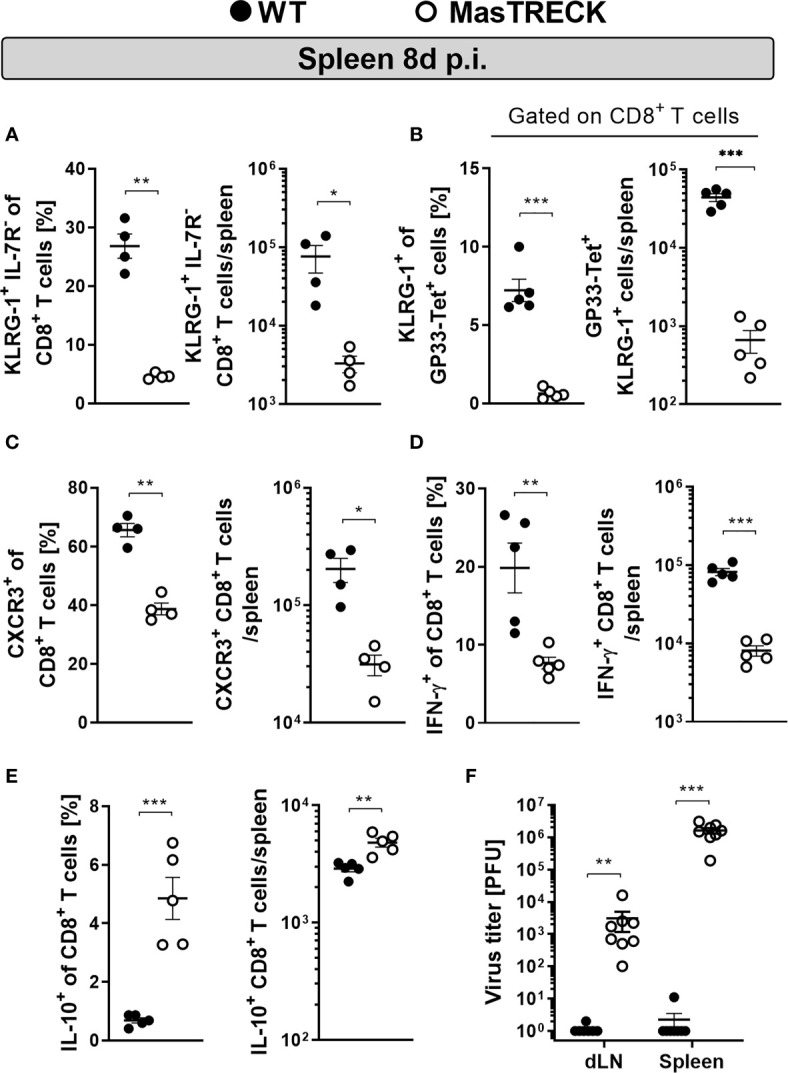
Mast cell deficient mice show impaired CD8*^+^* T cell effector phenotype and antiviral cytokine production after infection. Frequencies and absolute numbers of **(A)** KLRG1^+^ IL-7R^-^, **(B)** KLRG1^+^ gated on GP33-Tet^+^ CD8^+^ T cells and **(C)** CXCR3^+^ CD8^+^ T cells in the spleen of WT and MasTRECK mice at day 8 post intradermal LCMV infection. Frequency and absolute number of **(D)** IFN-γ– and **(E)** IL-10–producing CD8^+^ T cells in the spleen after *ex vivo* restimulation with GP33 and NP396 peptides of WT and MasTRECK mice at day 8 post LCMV infection analyzed by flow cytometry. **(F)** Virus titers in ear-dLNs and spleen of WT and MasTRECK mice assessed by plaque assay at day 8 post intradermal LCMV infection. All experiments were performed at least twice, and each experimental group included n ≥ 4. Data are representative and expressed as mean ± SEM. Statistically significant differences are analyzed by *t-* test and indicated as follows: *p < 0.05, **p < 0.01, ***p < 0.001.

We then characterized the phenotype and function of CD8***^+^*** T cells of WT and MasTRECK mice in the ear-dLN at day 5 post infection and in the spleen at the peak of LCMV infection (**Figure S2**). Uninfected WT and MasTRECK mice displayed similar frequencies of naïve (CD44***^-^*** CD62L***^+^*)** as well as activated CD8*^+^* T cells (CD44***^+^*** CD62L***^-^***) (**Figure S7A**) as well as comparable frequencies of IFN-γ–producing CD8*^+^* T cells in the spleen (**Figure S7B**). However, infected MasTRECK mice displayed markedly reduced frequencies and absolute numbers of short-lived effector CD8*^+^* T cells, depicted by KLRG-1*^+^* IL-7R*^-^* expression, compared to that of infected WT animals in the ear-dLNs (**Figure S8A**) and in the spleen (**Figure 3A** and **Figure S9A**). Furthermore, we observed that not only total CD8*^+^* T cells showed an impaired effector phenotype, but also the few GP33-tetramer^+^ CD8^+^ T cells found in the spleen of infected MasTRECK mice displayed markedly reduced KLRG-1 expression compared to that of GP33-tetramer^+^ CD8^+^ T cells of WT infected mice (**Figure 3B**). In addition, CD8*^+^* T cells that expressed CXCR3, shown to be up-regulated on the surface of activated CD8*^+^* T cells and important for their recruitment to antigen-rich areas of the spleen (40) were diminished in the ear-dLNs (**Figure S8B**) and in the spleen (**Figure 3C**) of infected MasTRECK mice compared to that of infected WT animals. Furthermore, infected MastTRECK mice had markedly reduced frequencies and absolute numbers of IFN-γ–producing CD8*^+^* T cells after GP33 and NP396 peptide restimulation *ex vivo* in ear-dLN (**Figure S8C**) and in the spleen (**Figure 3D** and **Figure S9B**) compared to that of WT animals. In contrast, the frequency and the absolute cell number of CD8*^+^* T cells producing the anti-inflammatory cytokine, IL-10 were increased in ear-dLNs (**Figure S8D**) and in the spleen (**Figure 3E**) of infected MasTRECK mice compared to that of WT mice.

In line with the impaired effector phenotype and defective cytokine production displayed by their virus-specific CD8^+^ T cells, MasTRECK mice were unable to control LCMV infection and exhibited significantly higher viral loads in the spleen and ear-dLNs at the peak of infection compared to that of infected WT mice (**Figure 3F**). These findings collectively suggest that after intradermal LCMV infection, MCs are essential for antigen-specific CD8*^+^* T cell effector differentiation, antiviral cytokine production and viral clearance at the peak of infection.

### Dendritic Cell Activation Is Impaired in Mast Cell Deficient Mice After LCMV Infection

**Figure 4:**
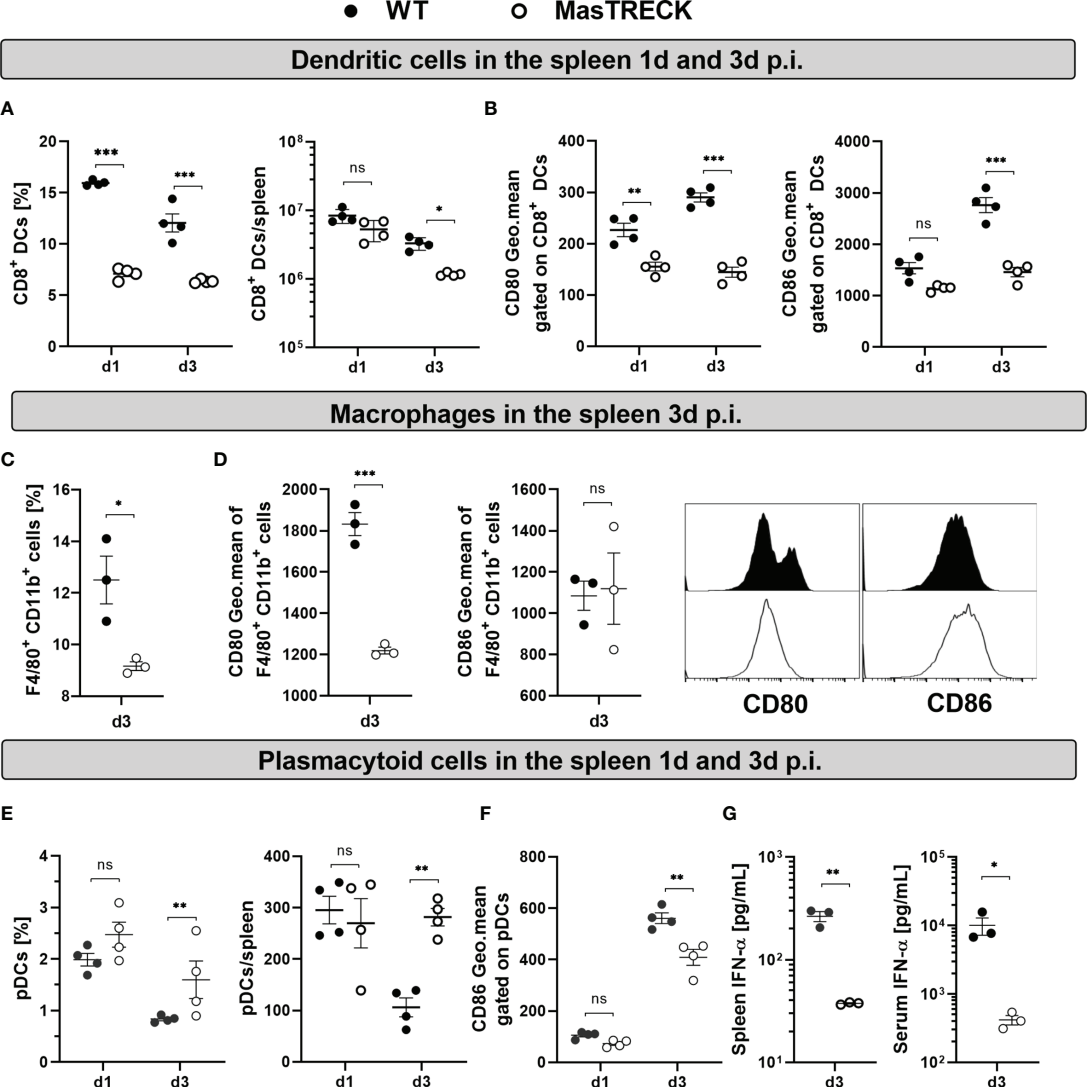
Dendritic cell activation is impaired in mast cell deficient mice after LCMV infection. **(A)** Frequency and absolute number of CD8^+^ DCs in the spleen of WT and MasTRECK mice on day 1 and 3 post intradermal LCMV infection. **(B)** Expression levels of CD80 and CD86 (geometric mean of fluorescence intensity) gated on CD8^+^ DCs in the spleen of WT and MasTRECK mice at day 1 and 3 post infection. **(C)** Frequency of splenic macrophages (F4/80^+^ CD11b^+^) and **(D)** expression levels of CD80 and CD86 (geometric mean of fluorescence intensity) gated on macrophages as well as representative histograms in the spleen of WT and MasTRECK mice at day 3 post infection. **(E)** Frequency and absolute number of pDCs in the spleen of WT and MasTRECK mice at day 1 and 3 post infection. **(F)** Expression levels of CD86 (geometric mean of fluorescence intensity) gated on pDCs in the spleen of WT and MasTRECK mice at day 1 and 3 post infection. **(G)** IFN-α concentration in the spleen and serum of WT and MasTRECK mice at day 3 post LCMV infection assessed by magnetic beads multiplex ELISA. All experiments were performed at least twice, and each experimental group included n ≥ 3. Data are representative and expressed as mean ± SEM. Statistically significant differences are analyzed by *t-* test and indicated as follows: ns = not significant, *p < 0.05, **p < 0.01, ***p < 0.001.

The initiation of antigen-specific CD8^+^ T cell responses requires the interaction of naive CD8^+^ T cells with mature DCs (41). Particularly, CD8^+^ DCs have been shown to be crucial in the initiation of CD8^+^ T cell responses after LCMV infection (42, 43) and also appeared to dominate cytotoxic T cell priming after skin infection (44). Since the virus-specific CD8*^+^* T cell immune response was strongly impaired in infected MasTRECK mice at the peak of the infection, we hypothesize that the absence of MCs hindered the proper activation of CD8^+^ DCs at early time points after LCMV infection. Therefore, we examined the frequency of CD8*^+^* DCs (CD8*^+^*MHCII*^+^*CD11c*^+^*) cells and their costimulatory molecule expression on in the spleen at day 1 and 3 post infection (**Figure S3A**). Infected MasTRECK mice exhibited reduced frequencies and absolute cell numbers of CD8*^+^* DCs compared to that of infected WT mice (**Figure 4A**). In addition, the mean fluorescent intensity of the costimulatory molecules CD80 and CD86 expressed on CD8*^+^* DCs was significantly lower in infected MasTRECK mice compared to that of WT mice at day 3 p.i.in the spleen (**Figure 4B**). Similarly, infected MasTRECK mice exhibited reduced frequencies and absolute cell numbers of CD8*^+^* DCs as well as reduced mean fluorescent intensity of CD86 in the ear-dLNs compared to that of infected WT mice (**Figures S10A, B**).

Splenic macrophages also play an important role in the activation of CD8^+^ T cells as well as in the control of viral load upon LCMV infection (45). Interestingly, infected MasTRECK mice exhibited reduced frequencies of F4/80*^+^* CD11b*^+^* cells (**Figure 4C**) and the mean fluorescent intensity of their costimulatory molecules CD80 but not CD86 was also significantly lower compared to that of WT mice at day 3 p.i. in the spleen (**Figure 4D**).

CD8*^+^* T cell activation is not only driven by properly activated DCs but also by the presence of pro-inflammatory cytokines such as type-I IFNs (46). Therefore, we assessed the frequency and activation of plasmacytoid DCs (pDCs) (**Figure S3B**) known to be the major producers of type-I IFNs (47). pDCs frequencies and absolute numbers were similar in the spleen of infected MasTRECK mice compared to that of WT animals at day 1 post infection but increased at day 3 p.i. (**Figure 4E**). Furthermore, the mean fluorescent intensity of CD86 on pDCs was significantly lower in infected MasTRECK mice compared to that of WT mice (**Figure 4F**). Interestingly, levels of IFN-α were also significantly reduced in the serum and in spleen homogenates of infected MasTRECK mice at day 3-post infection (**Figure 4G**). Taken together, these data indicate that the maturation of both classical and plasmacytoid DCs as well as the production of IFN-α after intradermal LCMV infection are impaired in the absence of MCs.

### Mast Cell Deficient Mice Display Decreased Chemokine Levels at the Peak of the Infection

**Figure 5:**
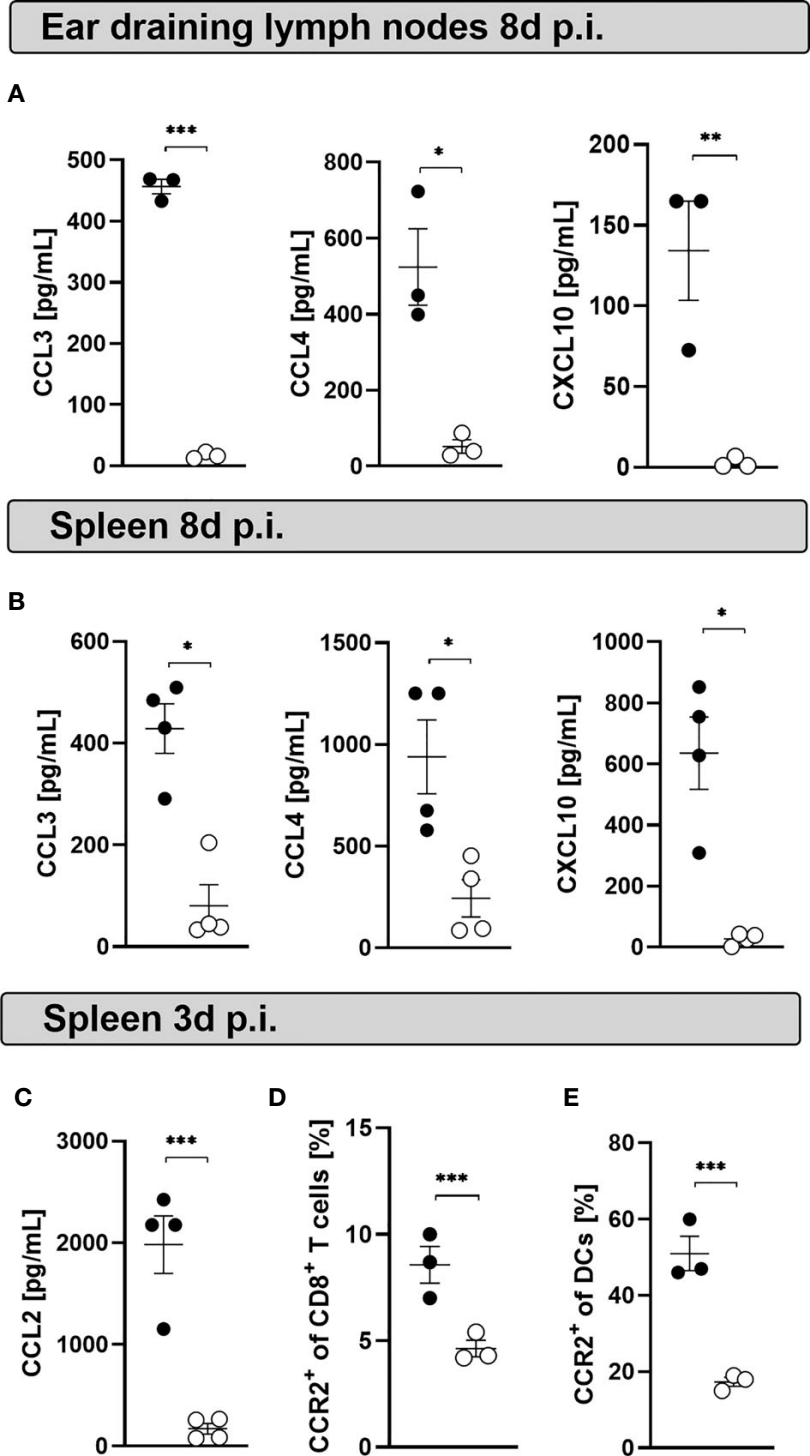
Mast cell deficient mice display decreased chemokine levels at the peak of the infection. Concentrations of CCL3, CCL4 and CXCL10 in the supernatants of cultured cells from **(A)** ear-dLNs and from the **(B)** spleen of WT and MasTRECK mice at day 8 post infection. **(C)** Concentrations of CCL2 in supernatants of cultured splenocytes of WT and MasTRECK mice at day 3 post infection assessed by the chemokine 26-Plex Mouse ProcartaPlex™ Panel 1. **(D)** Frequencies of CCR2^+^ CD8^+^ T cells and **(E)** CCR2^+^ DCs in the spleen of WT and MasTRECK mice at day 3 post infection analyzed by flow cytometry. All experiments were performed at least twice, and each experimental group included n ≥ 3. Data are representative and expressed as mean ± SEM. Statistically significant differences are analyzed by *t-* test and indicated as follows: *p < 0.05, **p < 0.01, ***p < 0.001.

MCs produce several cytokines and chemokines upon activation and during viral infections (48, 49). Chemokines such as CCL3 and CCL4 are produced by human cord blood-derived mast cells (CBMCs) infected with mammalian reovirus (50) and play a key role in T cell-DC interactions involved in the generation of immune responses (51). We hypothesized that MC deficiency induces a modified chemokine milieu that alters CD8*^+^* T cell and DC recruitment and activation. Indeed, CCL3, CCL4 and CXCL10 concentrations were decreased in ear-dLNs (**Figure 5A**) and spleen homogenates (**Figure 5B**) from infected MasTRECK mice compared to that of WT mice at day 8 p.i. Few frequencies of isolated peritoneal MCs (**Figure S4A**) were directly infected *in vitro* with LCMV at MOI 5 (**Figure S4B**). However, high levels of CXCL1 and CCL2 were detected in the supernatant of infected peritoneal MCs compared to uninfected counterparts after 24 hours p.i. (**Figures S4C, D**). Interestingly, infected MasTRECK mice displayed significantly lower concentrations of CCL2 in the spleen compared to that of infected WT mice at day 3 post LCMV infection (**Figure 5C**). CCR2, the receptor of CCL2 has been reported to play a critical role in the recruitment of DCs (52) and CD8*^+^* T cells during viral infections (53). Accordingly, infected MasTRECK mice exhibited lower frequencies of CCR2^+^ DCs and CCR2^+^ CD8*^+^* T cells in the spleen compared to that of WT mice at day 3 post LCMV infection (**Figures 5D, E**). These results show that the chemokine milieu in the secondary lymphoid organs is altered in the absence of MCs.

### Mast Cell Depletion After LCMV Infection Does Not Impair Virus-Specific CD8*^+^* T Cell Responses

**Figure 6:**
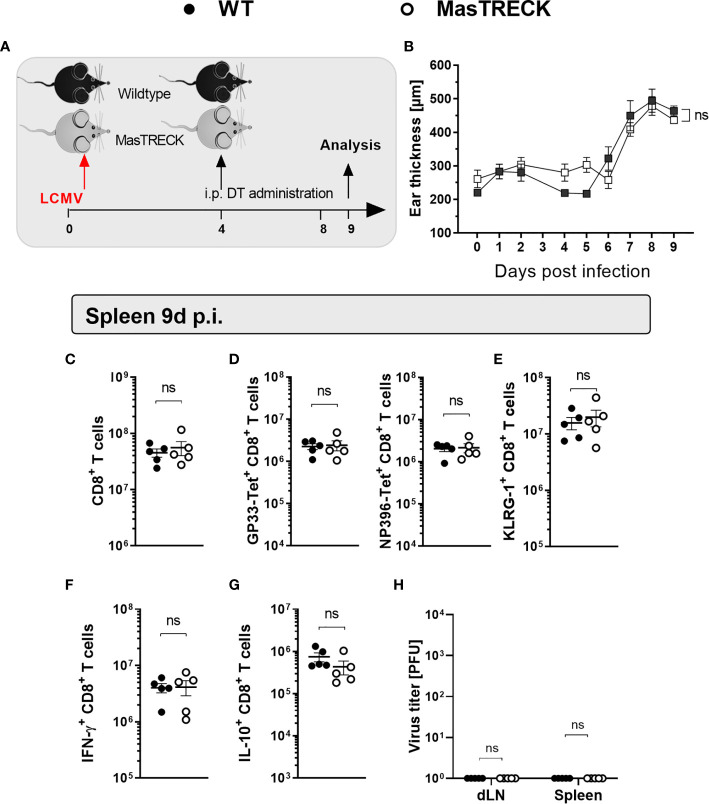
Mast cell depletion after LCMV infection does not impair virus-specific CD8+ T cell responses. **(A)** Schematic experimental layout to analyze virus-specific CD8^+^ T cell responses in WT and MasTRECK mice that were first infected with LCMV into the ventral side of the ear pinna and subsequently treated with 250 ng DT i.p. for 5 consecutive days between days 4-8 p.i. **(B)** Ear thickness daily measured using a caliper during 9 days after LCMV infection. **(C)** Absolute numbers of CD8^+^ T cells, **(D)** GP33-Tet^+^ and NP396-Tet^+^ CD8^+^ T cells as well as **(E)** KLRG1^+^ CD8^+^ T cells in the spleen of WT and MasTRECK mice at day 9 post infection. **(F)** Absolute numbers of IFN-γ– and **(G)** IL-10–producing CD8^+^ T cells in the spleen of WT and MasTRECK mice after *ex vivo* restimulation with GP33 and NP396 peptides at day 9 post infection assessed by flow cytometry. **(H)** Virus titers in ear-dLN and spleen of WT and MasTRECK mice assessed by plaque assay at day 9 post intradermal LCMV infection. All experiments were performed at least twice, and each experimental group included n ≥ 4. Data are representative and expressed as mean ± SD. Statistically significant differences are analyzed by *t*-test and indicated as follows: ns = not significant.

Our data suggested that the presence of MCs is important for proper DC maturation and recruitment at early time points after intradermal LCMV infection, which in turn is essential for proper effector differentiation, and antiviral cytokine production of antigen-specific CD8*^+^* T cells as well as viral clearance. Therefore, we investigated the impact of MC depletion on the phenotype and the function of virus-specific CD8*^+^* T cells at a later time point after LCMV infection. For this, WT and MasTRECK mice were initially infected with LCMV on the ventral side of the ear pinna, and DT treatment followed between day 4 and day 8 post infection (**Figure 6A**). Interestingly, the conditional depletion of mast cells at a later time point after infection completely reverted the suppression of LCMV-specific CD8^+^ T cell responses observed when MCs were depleted before the infection. No difference in the ear thickness was observed between infected WT and MasTRECK mice (**Figure 6B**). In addition, absolute cell numbers of CD8*^+^* T cells as well as GP33- and NP396-tetramer^+^ CD8^+^ T cells in the spleen of WT and MasTRECK mice were comparable at the peak of infection (**Figures 6C, D**). Furthermore, similar frequencies of KLRG1^+^ CD8^+^ T cells were observed in the spleen of both groups of infected animals (**Figure 6E**). Moreover, absolute cell numbers of IFN-γ– and IL-10–producing CD8*^+^* T cells in the spleen of infected MasTRECK mice after GP33 and NP396 peptide restimulation *ex vivo* were similar to that of infected WT mice (**Figures 6F, G**). As expected, both infected WT and MasTRECK mice were able to control LCMV infection and no viral loads were detected in the spleen and ear-dLNs at day 9 p.i. (**Figure 6H**). In summary, our data demonstrate that the presence of MCs at the beginning of the intradermal infection is crucial for the expansion, activation and antiviral cytokine production of virus-specific CD8*^+^* T cells.

## Discussion

In recent years, there is increasing evidence that MCs are key players of both innate and adaptive immune responses (1). MCs have been particularly known for their pivotal role in allergic type-I reactions (54). However, several studies have demonstrated that MC orchestrate the development of immune responses due to their strategic location, their ability to sense pathogens and danger as well as their capacity to directly and indirectly modify the activation and function of other immune cells (55). Furthermore, studies show MC influence on the cellular immune response to viruses (17). In a mouse model of Newcastle virus infection, recruitment of CD8^+^ T cells to the site of infection was dependent on the presence of MCs (56). MCs are equipped with a plethora of immune mediators that influence the migration, activation and function of granulocytes, DCs, macrophages, NK, NKT and T cells (24, 26, 48). We used transgenic MasTRECK mice that do not harbor any additional alterations of other immune cell subsets and it is an inducible model that allows us to deplete MCs with the administration of DT at different time points before and after LCMV infection. Although, basophils are also depleted after DT treatment, we do not envision any direct effects of basophils on antigen-specific CD8^+^ T cell responses upon LCMV infection known to induce a strong Th-1 immune response. Here, we report that MCs are crucial for the proper activation of DCs, splenic macrophages and pDCs during the first days of intradermal LCMV infection. The absence of MCs at the beginning of the intradermal LCMV infection led to an impaired activation, expansion and function of CD8^+^ T cells in infected mice.

The role of MCs in T cell expansion has been mostly associated with the ability of MCs to modulate DC migration to the dLNs (57, 58). In recent years, studies have revealed strong interactions between MCs and DCs that subsequently modulate their activation and their functionality. MC activation not only promotes DC migration (59) but also induces DC activation that in turn is critical for optimal CD8^+^ T cell activation (28). Accordingly, we show that in the absence of MCs, the frequency and absolute number of CD8^+^ DCs as well as their costimulatory molecules CD80 and CD86 were decreased during the first three days after intradermal LCMV infection. Subsequently, impaired DC activation led to the generation of defective virus-specific CD8^+^ T cells that displayed a dysfunctional effector phenotype characterized by low KLRG-1, low CXCR3 expression, reduced IFN-γ and high IL-10 production at the peak of infection that hindered the control of the viral clearance at the peak of the infection. In addition, the presence of MCs were also important for the proper activation of splenic macrophages. The presence of MCs at the beginning of the intradermal infection was crucial for the generation of optimal antigen-specific CD8^+^ T cells since MC depletion at day 4 post infection did not impair the phenotype or antiviral cytokine production of virus-specific CD8^+^ T cells and infected mice were able to control the infection.

One of the most challenging aspects for the role of MCs in the development of virus-specific T cell responses is the spatial separation between peripheral MCs and the CD8^+^ T cells in the spleen. However, MCs are not only located in the connective and mucosal tissue, they are also distributed around blood vessels and in close proximity to perivascular DCs (33). In addition, MCs can modulate CD8^+^ T cell responses over a distance, and signals from MCs can reach the spleen *via* the bloodstream, e.g. in the context of degranulation or *via* the release of the exosomes (60). Recent studies indicate that MCs can exert long distance effects through MC granule trafficking *via* lymphatic vessels and active shuttling of MC granules by DCs (61). Moreover, MCs can modulate T cell activation through exosomes that harbor inflammatory mediators (62, 63). CCL3 and CCL4 have been shown to facilitate T cell-DC interactions (51). In our study, the levels of CCL2, CCL3, CCL4 and CXCL10 were reduced in infected MC-deficient mice. We used peritoneal MCs as a surrogate for skin-MCs. Although very few frequencies of LCMV infected peritoneal MC were observed *in vitro* after 24 h p.i., high production of CCL2 and CXCL1 was detected. MCs could sense LCMV particles and produce chemokines such as CCL2 as previously reported (26). Furthermore, frequencies of CD8^+^ T cells–expressing CXCR3, the receptor for CXCL10, and of CD8^+^ T cells–expressing CCR2, the receptor for CCL2, were reduced in MC-deficient mice.

Collectively, our results indicate that after intradermal LCMV infection, MCs promote optimal CD8^+^ DC and pDC activation leading to the generation of a proper proinflammatory cytokine and chemokine milieu essential for the activation of antigen-specific CD8^+^ T cells that are crucial to achieve the control of the viral infection.

## Data Availability Statement

The original contributions presented in the study are included in the article/**Supplementary Material**. Further inquiries can be directed to the corresponding author.

## Ethics Statement

Animal protocols were performed in accordance with the German law for animal protection and the institutional guidelines of the Charité Berlin. The animal study was reviewed and approved by Landesamt fuer Gesundheit und Soziales LAGeSo G0078/17.

## Author Contributions 

FS, MMu, and MMa designed the research. YH and MMu performed the research. YH and MMu analyzed the data. YH, ML, and MMu performed the visualization, preparation, and presentation of the data. YH and MMu wrote the paper. All authors contributed to the article and approved the submitted version.

## Funding

The German Research Foundation (MU 4006/2-1) supported this work. MMu is a BIH‐Charité Clinical Scientist funded by the Charité - Universitätsmedizin Berlin and the Berlin Institute of Health.

## Conflict of Interest

The authors declare that the research was conducted in the absence of any commercial or financial relationships that could be construed as a potential conflict of interest.
