# Supplementary material for: Mast Cells Modulate Antigen-Specific CD8+ T Cell Activation During LCMV Infection
Source: Front Immunol. 2021 Jun 14;12:688347. doi: 10.3389/fimmu.2021.688347 (PMC8236703; doi:10.3389/fimmu.2021.688347)
Supplement: Supplementary file 1 [file DataSheet_1.pdf]

Supplementary Material

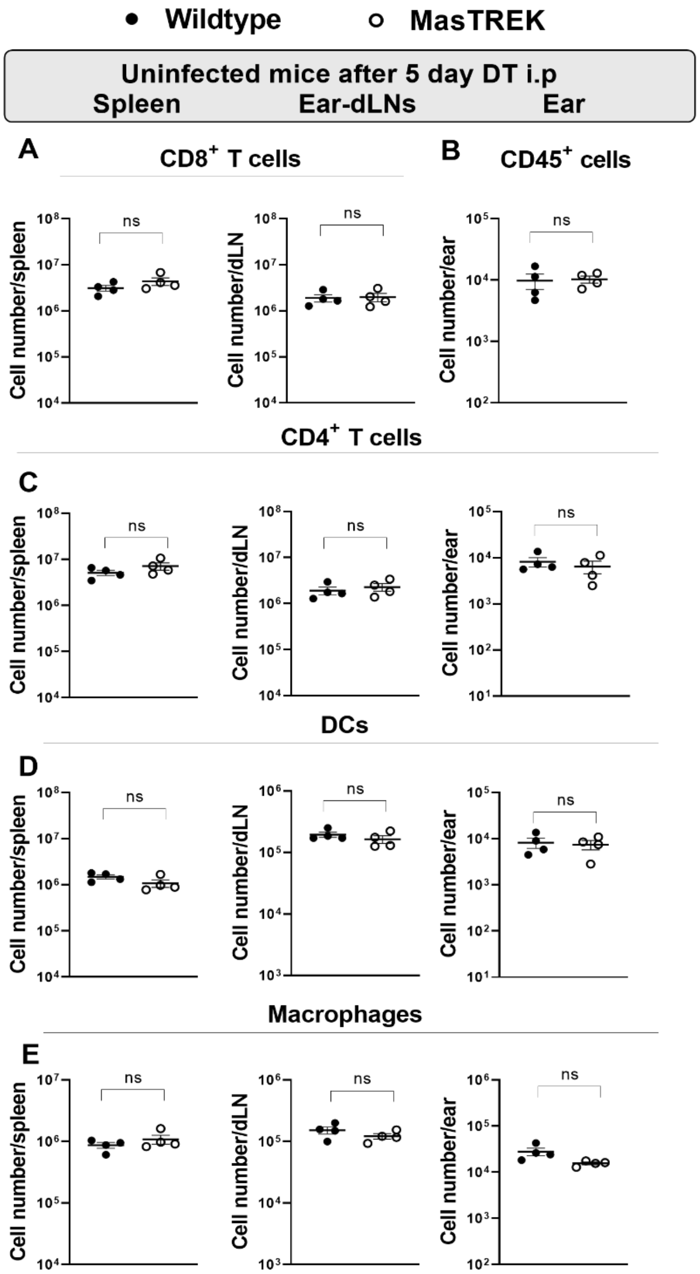

Supplementary Figure 1. Uninfected WT and MasTRECK mice exhibit similar frequencies of immune cells after MC depletion. (A) Absolute cell numbers of $CD8^+$ T cells in the spleen and dLN, (B) $CD45^+$ cells in the ears as well as (C) $CD4^+$ T cells, (D) DCs ($CD11c^+$ $MCHII^+$) and (E) macrophages ($F4/80^+$ $CD11b^+$) in the spleen, ear-dLN and ears of WT and MasTRECK mice treated on 5 consecutive days with DT (250 ng/mouse i.p.) before LCMV infection and analyzed by flow cytometry. All experiments were performed at least twice, and each experimental group included $n \geq 4$. Data are representative and expressed as mean ± SEM. Statistically significant differences are analyzed by t-test and indicated as follows: ns = not significant**.**

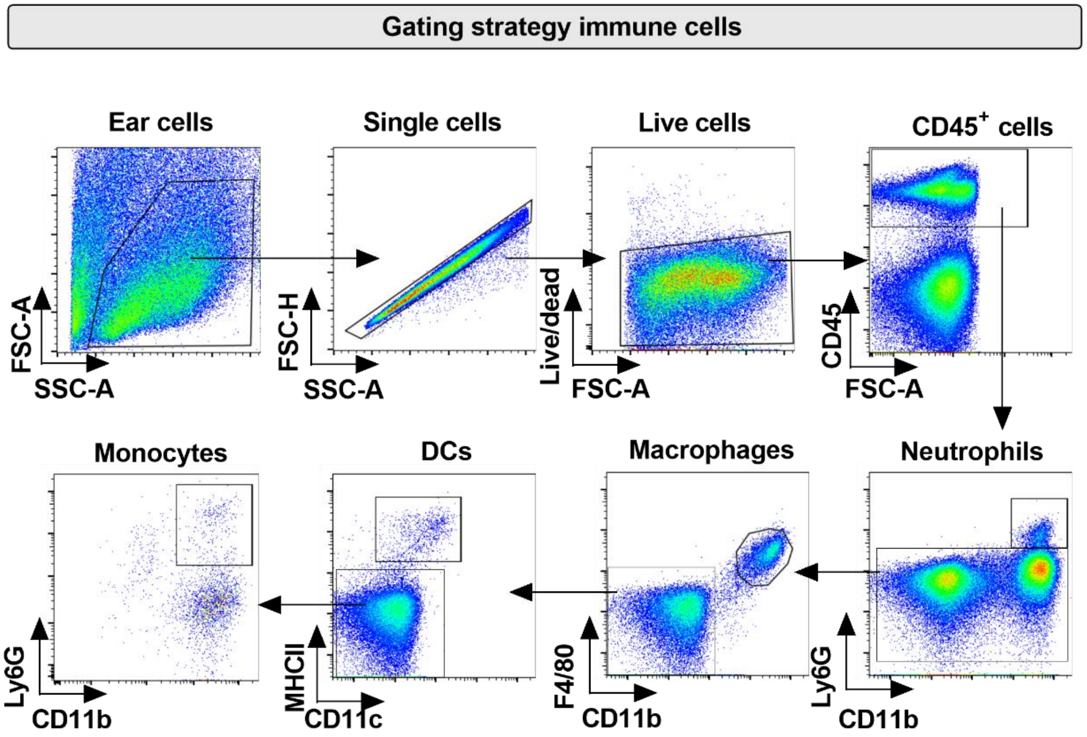

Supplementary Figure 2. Gating strategy for immune cell analysis.
Representative dot plots of the ear showing the gating strategy used for the identification of immune cell subsets in the spleen, ear-dLNs and ears of WT and MasTRECK mice. After gating the total immune cells followed by single cell gate, cells were gated on Zombie-Aqua$^-$ to discriminate between live and dead. Live cells were gated on haemotopoietic cells ($CD45^+$) that were subsequently gated on neutrophils (Ly6G $^+CD11b^+$), macrophages (F4/80 $^+CD11b^+$), DCs MHCII $^+CD11c^+$) and monocytes ($Ly6C^+CD11b^+$).

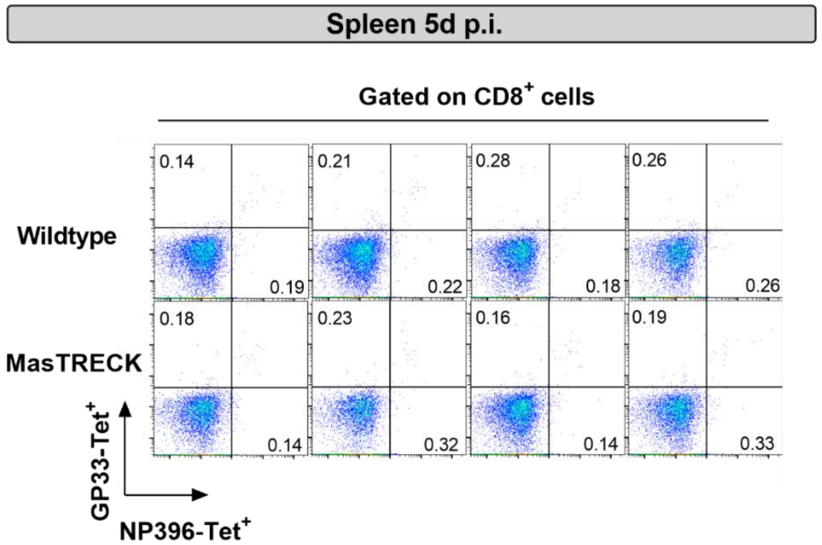

Supplementary Figure 3. Virus-specific $CD8^+$ T cells are not present in the spleen at day 5 post infection
FACS plots of GP33-$Tetramer^+$ and NP396-$Tetramer^+$ $CD8^+$ T cells in the spleen of infected WT and MasTRECK mice at day 5 post intradermal LCMV infection.

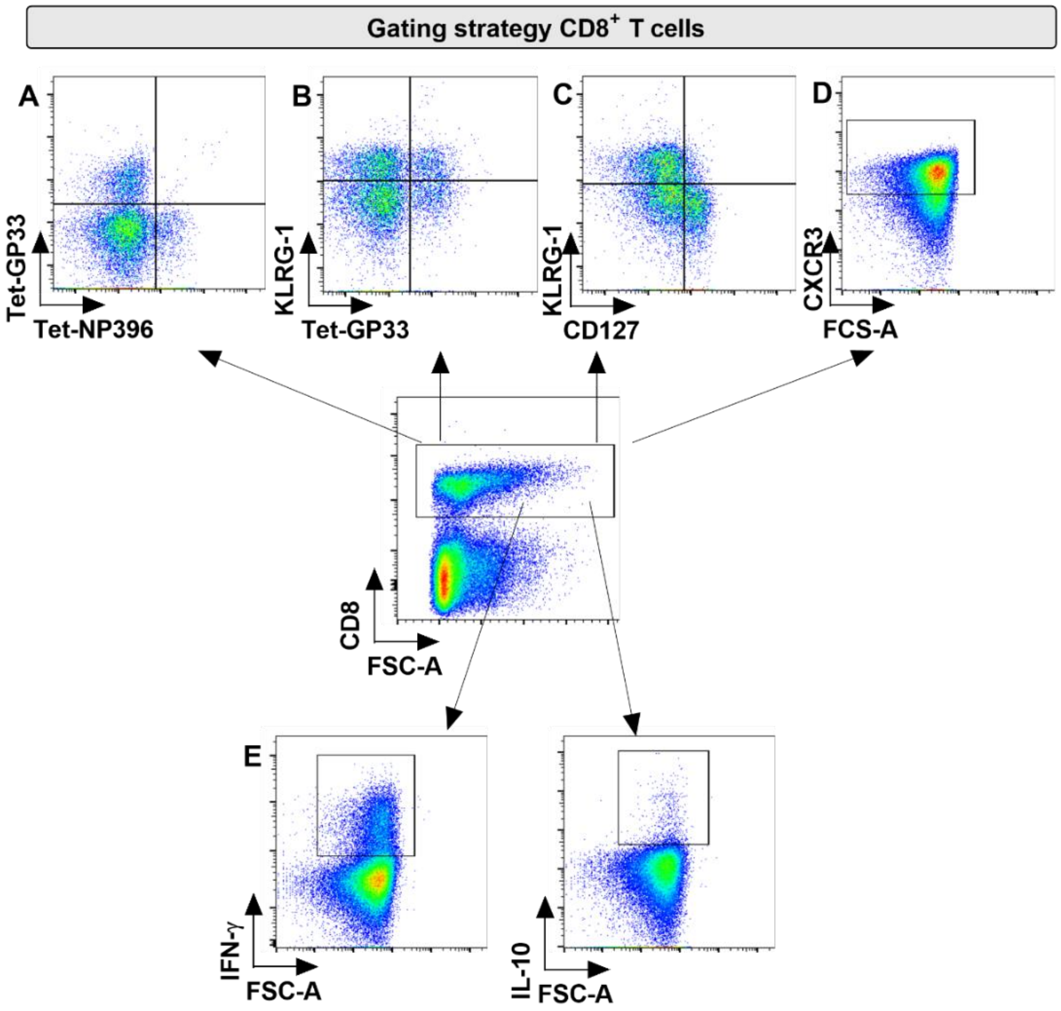

***Supplementary Figure 4. Gating strategy for CD8 T cells⁺ analysis**.*

Representative dot plots of the spleen showing the gating strategy used to identify and characterize $CD8^+$ T cells in the spleen, ear-dLNs and ears of WT and MasTRECK mice. After gating on $CD8^+$ T cells, LCMV-specific $CD8^+$ T cells were subsequently gated on (A) Tet-$GP33^+$ and Tet-$NP396^+$ and on (B) KLRG-1^+ Tet-$GP33^+$, short lived-effector $CD8^+$ T cells were gated on (C) KLRG-1^+ IL-$7R^-$ and (D) $CXCR3^+$ and cytokine producing cells were gated on (E) IFN-γ^+ and IL-10^+.

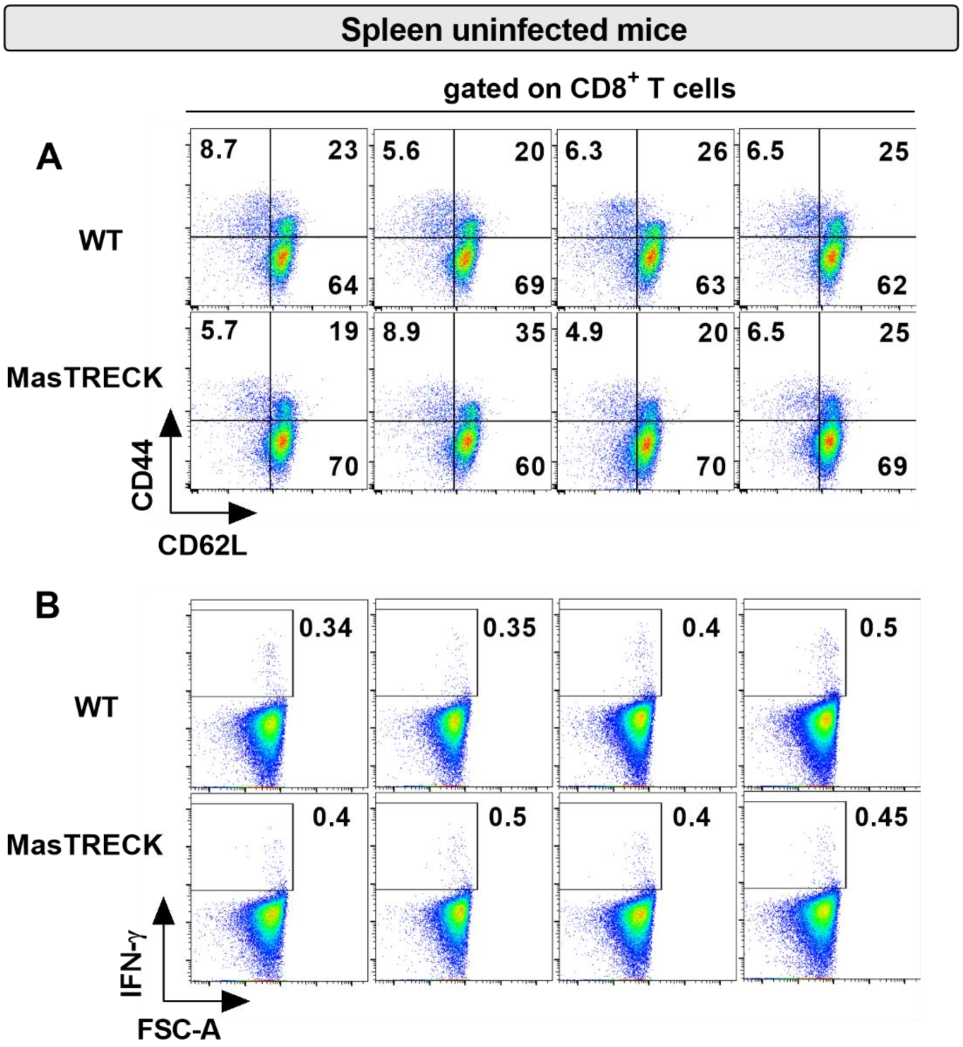

***Supplementary Figure 5**.* **Uninfected WT and MC-deficient mice show comparable frequencies of naïve, activated and IFN-γ–producing CD8$^+$ T cells.**

FACS plots show frequencies of (A) activated (CD44$^+$ CD62$^-$) and naïve (CD44$^-$ CD62$^+$) CD8$^+$ T cells and (B) IFN-γ–producing CD8$^+$ T cells in the spleen after *ex vivo* restimulation with PMA/Ionomycin of uninfected WT and MasTRECK mice.

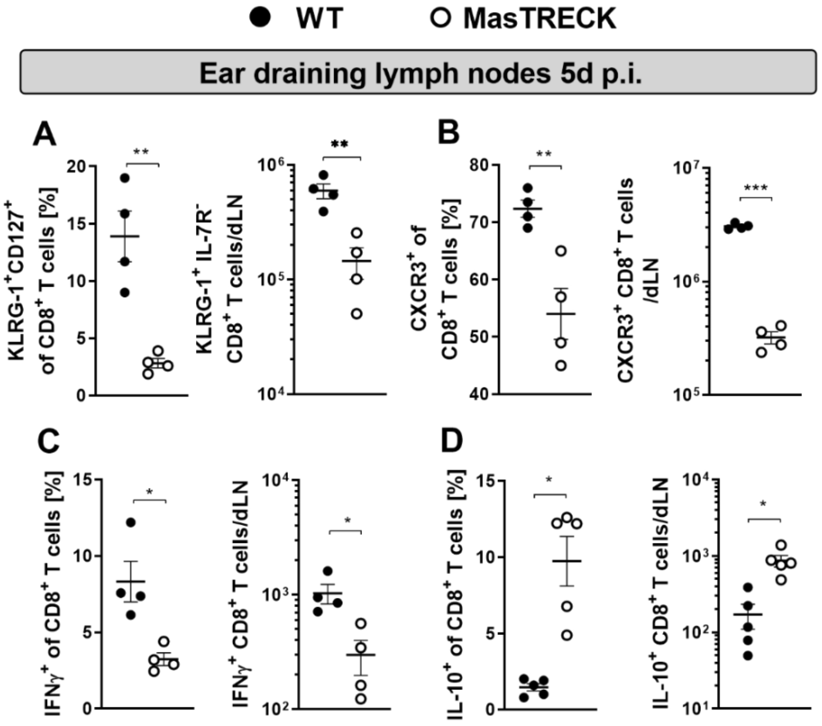

***Supplementary Figure 6. Mast cell deficient mice show impaired CD8+ T cell phenotype and defective antiviral cytokine production in the ear*-dLN** (A) Frequency and absolute number of KLRG1+ IL-7R- cells and (B) CXCR3+ gated on CD8+ T cells in the ear-dLNs of WT and MasTRECK mice at day 5 post infection. (C) Frequencies and absolute numbers of IFN-γ– and (D) IL-10–producing CD8+ T cells in the ear-dLNs of WT and MasTRECK mice after ex vivo restimulation with GP33 and NP396 peptides. All experiments were performed at least twice, and each experimental group included n ≥ 4. Data are representative and expressed as mean ± SEM. Statistically significant differences are analyzed by t-test and indicated as follows: *p < 0.05, **p < 0.01, ***p < 0.001.

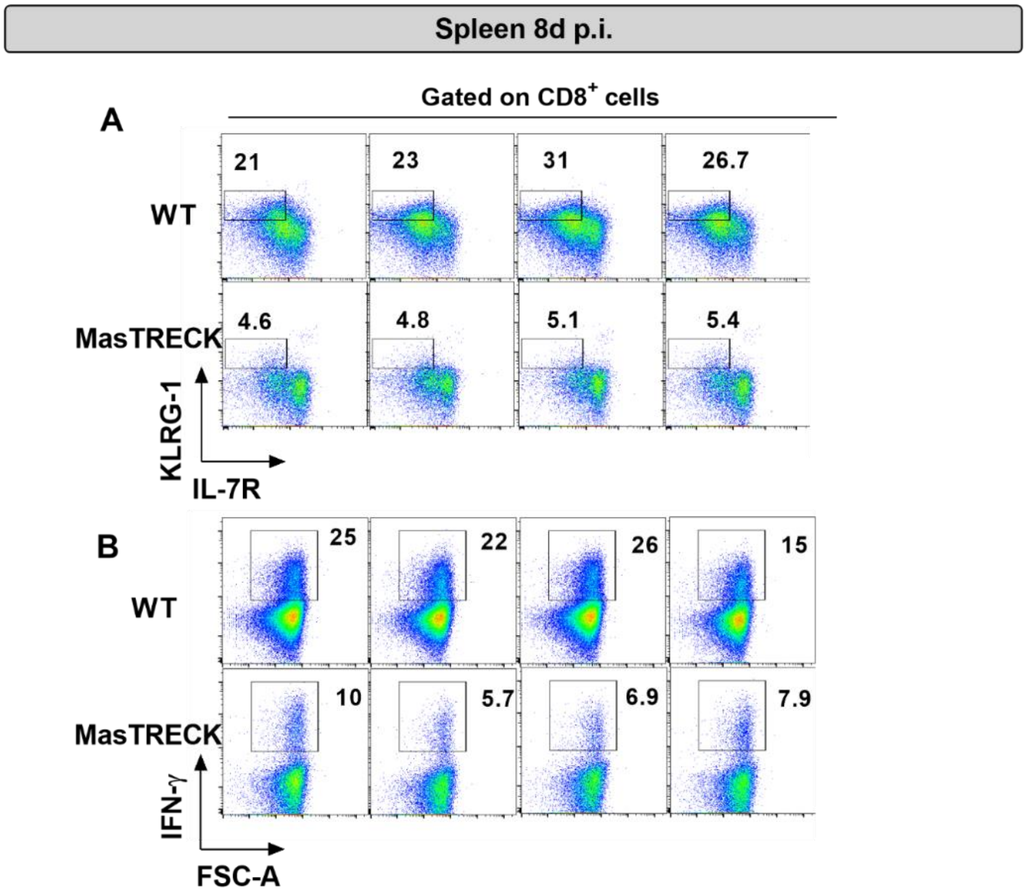

Supplementary Figure 7. **Mast cell deficient mice show impaired $CD8^+$ T cell effector phenotype and antiviral cytokine production after infection**

FACS plots show frequencies of (A) $KLRG1^+$ $IL\text{-}7R^-$ $CD8^+$ T cells and IFN.γ–producing $CD8^+$ T cells in the spleen after *ex vivo* restimulation with GP33 and NP396 peptides of WT and MasTRECK mice at day 8 post LCMV infection.

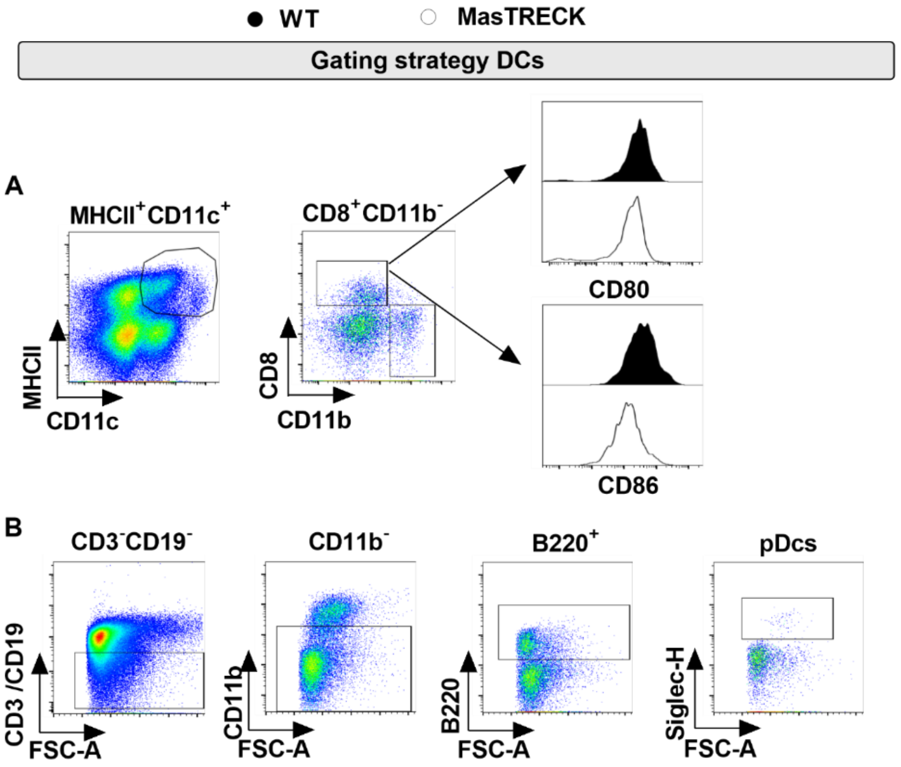

Supplementary Figure 8. Gating strategy for DCs analysis
Representative dot plots of the spleen showing the gating strategy used to analyze (A) DCs and (B) pDCs in the spleen, ear-dLNs and ears of WT and MasTRECK mice. After gating on DCs (MHCII$^+$CD11c$^+$), lymphocytic DCs were gated on CD8$^+$ CD11b$^-$ cells. Geometric mean fluorescent intensity of costimulatory molecules CD80 and CD86 was subsequently analyzed on CD8$^+$ CD11b$^-$ DCs as shown in the representative histograms. pDCs are Siglec-H$^+$ cells gated on CD11b$^-$ cells that were previously gated on CD3$^-$ CD19$^-$ CD11b$^-$ cells.

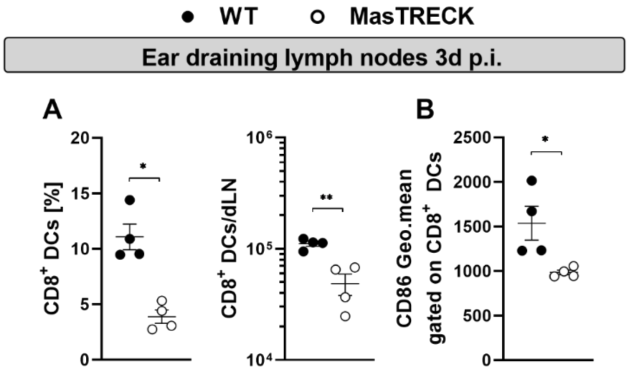

Supplementary Figure 9. Dendritic cells activation is impaired in the ear-dLN after LCMV infection in mast cell deficient mice (A) Frequency and absolute number of CD8$^+$ DCs in the dLNs of WT and MasTRECK mice on day 3 post LCMV infection. (B) Expression levels of CD86 (geometric mean of fluorescence intensity) gated on CD8$^+$ DCs in the spleen of WT and MasTRECK mice at day 3 post infection. All experiments were performed at least twice, and each experimental group included $n \geq 4$. Data are representative and expressed as mean ± SEM. Statistically significant differences are analyzed by t-test and indicated as follows: $*p < 0.05$, $**p < 0.01$.

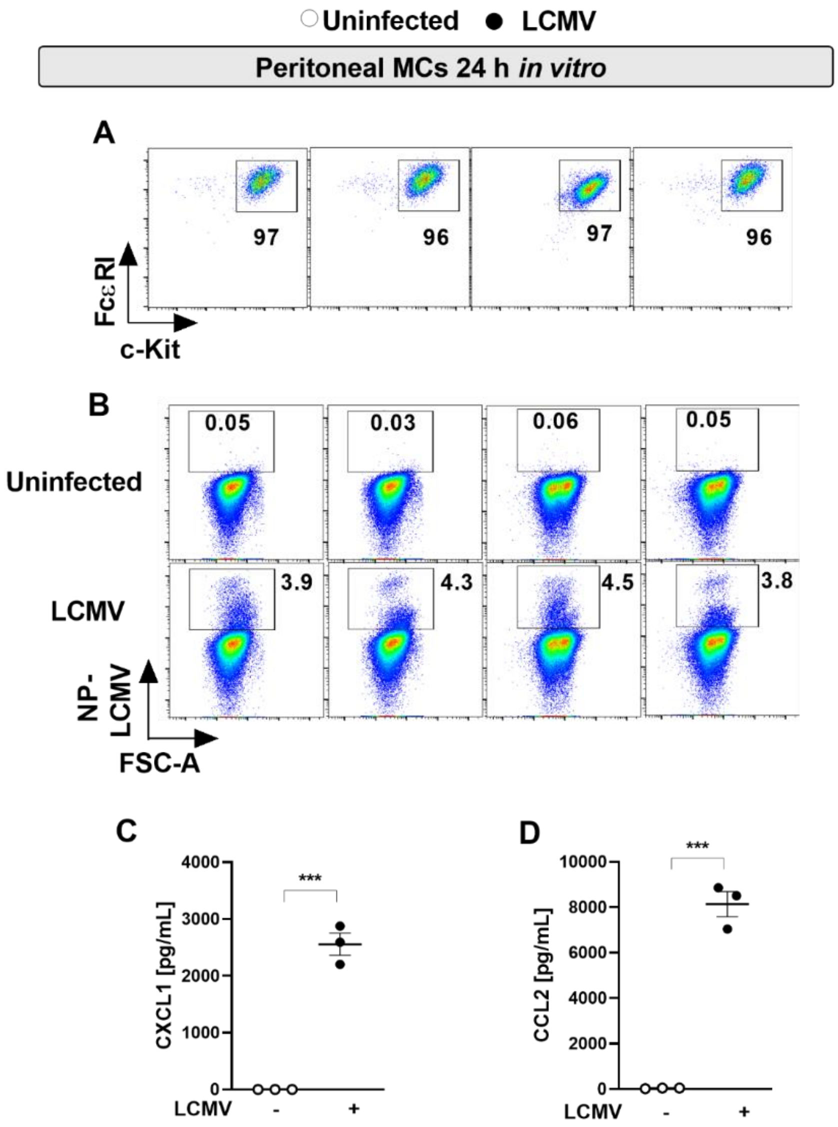

***Supplementary Figure 10**. MCs produce CXCL1 and CCL2 after LCMV infection in vitro*

*(*A) FACS plots show peritoneal MCs (FcεRI⁺ c-Kit⁺) after isolation by flow cytometry. (B) FACS plots show frequencies of peritoneal MCs infected with LCMV at MOI 5 for 24h *in vitro*. (C) CXCL1 and (D) CCL2 concentrations in the supernatant of peritoneal murine MCs after 24h of LCMV infection (MOI 5) assessed by the chemokine 26-Plex Mouse ProcartaPlex™ Panel 1.

All experiments were performed at least twice, and each experimental group included $n \geq 3$. Data are representative and expressed as mean ± SEM. Statistically significant differences are analyzed by t-test and indicated as follows: ***$p < 0.001$.
